# Hypoxia-inducible factor-1 alpha maintains mouse articular cartilage through suppression of NF-κB signaling

**DOI:** 10.1038/s41598-020-62463-4

**Published:** 2020-03-25

**Authors:** Keita Okada, Daisuke Mori, Yuma Makii, Hideki Nakamoto, Yasutaka Murahashi, Fumiko Yano, Song Ho Chang, Yuki Taniguchi, Hiroshi Kobayashi, Hiroaki Semba, Norihiko Takeda, Wen Piao, Kenjiro Hanaoka, Tetsuo Nagano, Sakae Tanaka, Taku Saito

**Affiliations:** 1 Sensory & Motor System Medicine, The University of Tokyo, 7-3-1 Hongo, Bunkyo-ku, Tokyo 113-8655 Japan; 2 Bone and Cartilage Regenerative Medicine, The University of Tokyo, 7-3-1 Hongo, Bunkyo-ku, Tokyo 113-8655 Japan; 3 Department of Cardiovascular Medicine, Graduate School of Medicine, The University of Tokyo, 7-3-1 Hongo, Bunkyo-ku, Tokyo 113-8655 Japan; 4 Graduate School of Pharmaceutical Sciences, The University of Tokyo, 7-3-1 Hongo, Bunkyo-ku, Tokyo 113-8655 Japan; 5 Drug Discovery Initiative, The University of Tokyo, 7-3-1 Hongo, Bunkyo-ku, Tokyo 113-8655 Japan

**Keywords:** Mechanisms of disease, Osteoarthritis

## Abstract

HIF-1α, an essential transcription factor under hypoxic condition, is indispensable for chondrocytes during skeletal development but its expression and roles in articular chondrocytes are yet to be revealed. We examined HIF-1α protein expression and the hypoxic condition during mouse osteoarthritis (OA) development using state of the art hypoxic probes and found that its expression decreased as OA progressed, coinciding with the change in hypoxic conditions in articular cartilage. Gain- and loss-of-function of HIF-1α in cell culture experiments showed that HIF-1α suppressed catabolic genes such as *Mmp13* and *Hif2a*. We confirmed these anticatabolic effects by measuring glycosaminoglycan release from wild type and conditional knock-out mice femoral heads cultured *ex vivo*. We went on to surgically induce OA in mice with chondrocyte-specific deletion of *Hif1a* and found that the development of OA was exacerbated. Increased expression of catabolic factors and activation of NF-κB signalling was clearly evident in the knock-out mice. By microarray analysis, C1qtnf3 was identified as a downstream molecule of HIF-1α, and experiments showed it exerted anti-catabolic effects through suppression of NF-κB. We conclude that HIF-1α has an anti-catabolic function in the maintenance of articular cartilage through suppression of NF-κB signalling.

## Introduction

Advancement in the field of musculoskeletal research has resulted in unveiling molecular mechanisms of diseases such as osteoporosis, osteoarthritis (OA) and rheumatoid arthritis (RA). These have led to inventions of evolutionary treatment and the effects have significantly decreased the number of patients requiring surgical treatment in RA. OA is a multifactorial entity caused by mechanical stress and inflammation, so to reproduce the mechanism experimentally was challenging. However, since the introduction of murine surgical knee OA models, understanding of the molecular mechanisms and signalling pathways of the disease has accelerated. Matrix metalloproteinase-13 (Mmp3), nuclear factor kappa B (NF-κB), a disintegrin-like and metallopeptidase with thrombospondin type 1 motif 5 (Adamts5) and hypoxia-inducible factor 2-alpha (HIF-2α) are the representative catabolic factors revealed to date^1–10^. Among them, HIF-2α is an essential transcription factor in the pathophysiology of OA. HIF-2α is a member of HIF regulatory α-subunit proteins^11^ and is actually abundantly expressed in intermediate and deep layers of osteoarthritic cartilage^8^. HIF-2α is a direct transcriptional target of NF-κB, and its expression is dependent on the activation of NF-κB signalling by various stimulations^8,10^. Increased HIF-2α protein further induces various catabolic factors including Mmp13, which subsequently accelerate cartilage degeneration^8^.

Besides HIF-2α, HIF-1α is a representative member of the HIF family. HIF-1α proteins only exist under hypoxic conditions, and exert cyto-protective effects. Both HIF proteins share approximately 50% amino acid homology^12^; however, in contrast to HIF-2α, HIF-1α plays essential roles in development of cartilage, which is avascular and exists under hypoxic condition^13^. During limb formation, HIF-1α is dispensable for condensation of limb bud mesenchyme, but required for early chondrocyte differentiation^14^. HIF-1α is also indispensable for normal joint development, and chondrocyte survival in the growth plate^13,14^. In spite of these previous findings of HIF-1α in differentiating chondrocytes, the roles of HIF-1α and the association of HIF-1α and HIF-2α in articular cartilage have not been fully understood.

Considering the anabolic roles of HIF-1α in epiphyseal chondrocytes during skeletal development, we hypothesized that HIF-1α may exert some effects against HIF-2α and be involved in homeostasis of articular cartilage or OA development. The present study aimed to reveal roles of HIF-1α in cartilage degeneration, particularly in association with the catabolic effects of the NF-κB - HIF-2α pathways. Here, we examined expression of HIF-1α protein during articular cartilage degeneration, and its roles by mouse primary chondrocytes, femoral head culture, and surgical OA model using HIF-1α conditional knockout mice. We further analysed molecular mechanisms underlying the effects of HIF-1α by a microarray analysis and *in vitro* experiments.

## Results

### Expression of HIF-1α protein during OA development

**Figure 1:**
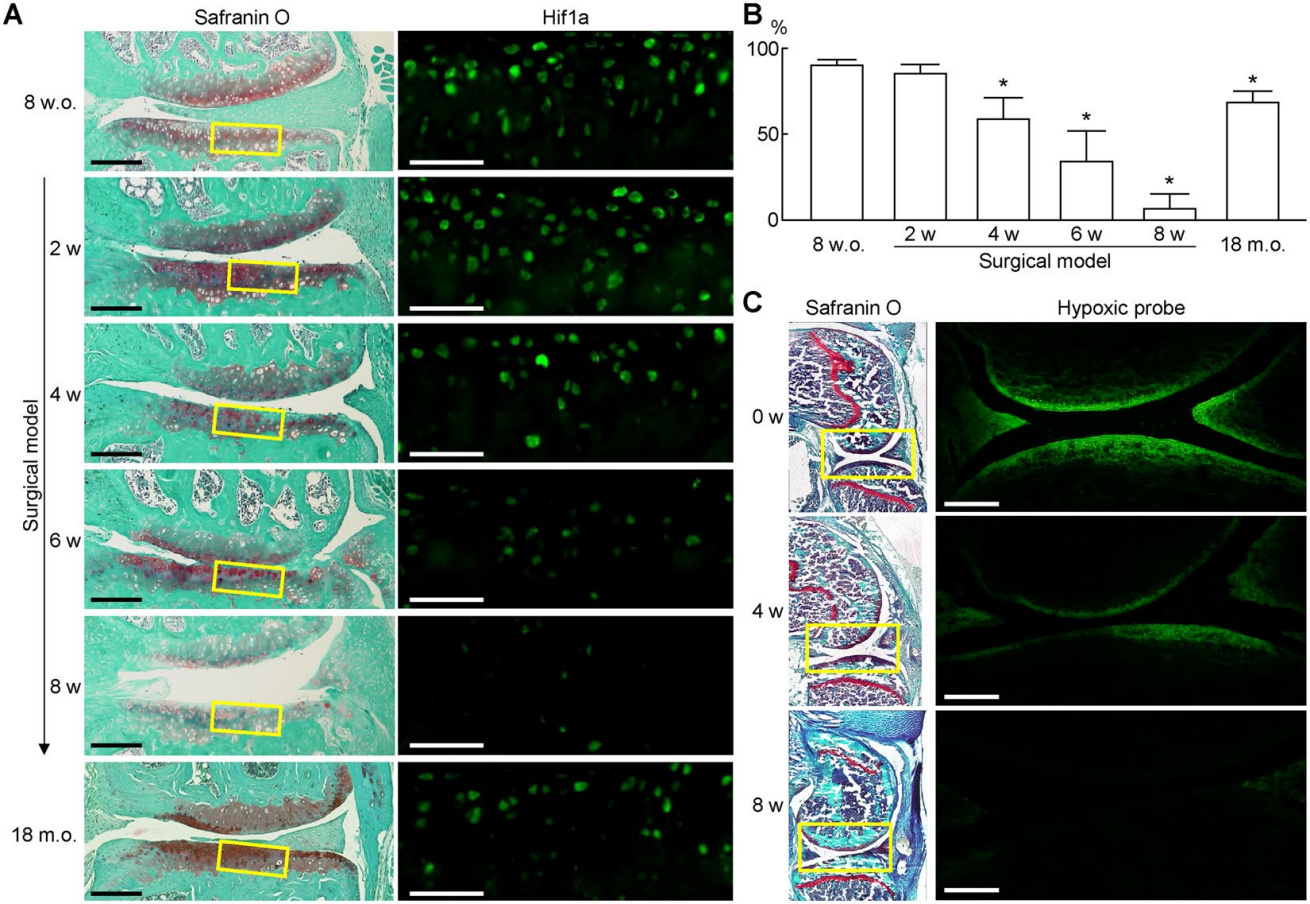
Expression of HIF-1α protein during OA development. (**A**) Safranin O staining and immunofluorescence of HIF-1α in mouse knee cartilage. From top to bottom, 8 weeks old (8 w.o.: before OA surgery), 2, 4, 6, and 8 weeks after surgery, and 18 months old (18 m.o.) without surgical induction. Inset boxes in middle immunofluorescence images indicate the regions of high magnification. Scale bars, 100 µm and 50 µm for low and high magnification images, respectively. (**B**) The percentage of HIF-1α positive cells in the immunofluorescence. *P < 0.05 vs 0 w. (**C**) Fluorescent images of the hypoxic probe in mouse knee cartilage before surgery (0 w), 4 and 8 weeks after surgery. The mice were sacrificed five hours after intraarticular injection of the hypoxic probe, and analysed by frozen section technique. Inset boxes in Safranin O staining indicate the regions of fluorescent images of the hypoxic probe. Scale bars, 200 µm.

We initially examined expression of HIF-1α protein in mouse surgically-induced OA models. In 8-week-old wild-type (WT) mouse knee joint, abundant expression of HIF-1α protein was detected in the articular cartilage (Fig. 1A). The rate of HIF-1α-positive cells decreased from 4 to 8 weeks after OA induction (Fig. 1A,B). Expression of HIF-1α protein in 18-month-old cartilage was similar to that of 4 weeks after OA induction (Fig. 1A,B). To examine alteration of oxygen concentration in articular cartilage with OA development, we used an Azo-based fluorescent hypoxic probe MAR^15^. We first tested the MAR using mouse primary chondrocytes. The cells on a culture plate were treated with the MAR, and sealed with a round cover glass (Supplementary Fig. S1A). After one hour culture, the signal intensity was detected around the centre of the cover glass (Supplementary Fig. S1B), indicating that the MAR functioned properly in chondrocytes. We then injected the MAR into WT mice knee joints before the OA surgery, 4 and 8 weeks after the surgery, and sacrificed them after five hours. The fluorescent signal was clearly detected in the surface of articular cartilage and menisci of the normal knee joints (Fig. 1C). However, the signal was decreased at 4 weeks, and hardly detected at 8 weeks after the surgery (Fig. 1C). These data indicate that loss of hypoxic condition and decreased expression of HIF-1α protein may be associated with OA development.

### Regulation of catabolic genes by HIF-1α

**Figure 2:**
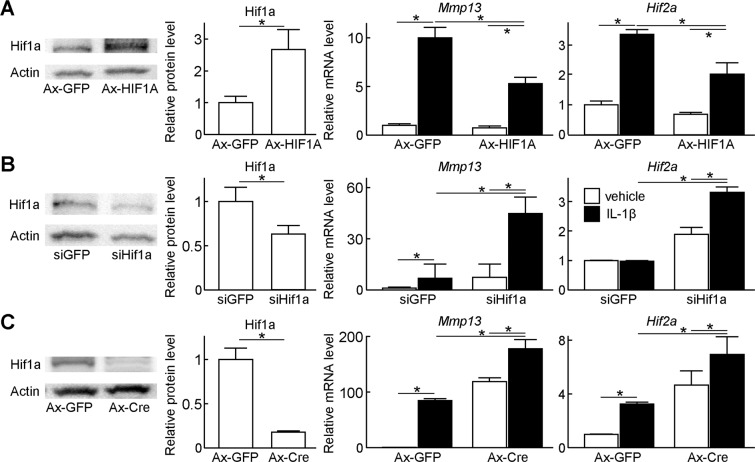
Regulation of catabolic genes by HIF-1α. (**A**) Protein levels of Hif1a, and mRNA levels of *Mmp13*, and *Hif2a* in WT mouse primary chondrocytes transduced with GFP or HIF1A adenoviral vectors under the hypoxic condition (3% O_2_). GFP or HIF1A was transduced at a multiplicity of infection (MOI) of 100. The cells were treated with or without 10 ng/mL IL-1β for 2 days. Bars show the mean ± SD of three samples per group. *P < 0.05. (**B**) Protein levels of Hif1a, and mRNA levels of *Mmp13*, and *Hif2a* in WT mouse primary chondrocytes transfected with siRNA against *GFP* or *Hif1a* under the hypoxic condition. The cells were treated with or without 10 ng/mL IL-1β for 2 days. Bars show the mean ± SD of three samples per group. *P < 0.05. (**C**) Protein levels of Hif1a, and mRNA levels of *Mmp13*, and *Hif2a* in *Hif1a*^*fl/fl*^ primary chondrocytes transduced with GFP or Cre adenoviral vectors under the hypoxic condition. GFP or Cre was transduced at a MOI of 100. The cells were treated with or without 10 ng/mL IL-1β for 2 days. Bars show the mean ± SD of three samples per group. *P < 0.05.

We next investigated association of chondrocyte catabolism and HIF-1α by cell culture experiments. For a gain-of-function study, we transduced adenoviral vectors of HIF1A or GFP into WT mouse primary chondrocytes (Fig. 2A). In GFP control cells, IL-1β treatment markedly induced Mmp13, a representative catabolic enzyme for cartilage matrix; however, in HIF-1α overexpressing cells, the induction of *Mmp13* was significantly suppressed (Fig. 2A). *Hif2a*, a potent upstream factor of Mmp13, was also increased by IL-1β, but suppressed by HIF-1α overexpression (Fig. 2A). For a loss-of-function study, we transfected siRNA against Hif1a or GFP into WT mouse primary chondrocytes. Although the knockdown efficiency of Hif1a protein was approximately 50%, induction of *Mmp13* and *Hif2a* by IL-1β treatment was significantly enhanced in *Hif1a*-suppressed cells compared with those in control cells (Fig. 2B). We further transduced adenoviral vectors of Cre recombinase or GFP into primary chondrocytes derived from *Hif1a*^*fl/fl*^ mice. Hif1a protein level in Cre-transduced cells was decreased to 17.7% of that in GFP-transduced control cells (Fig. 2C). Subsequently, induction of *Mmp13* and *Hif2a* by IL-1β treatment was significantly enhanced in *Hif1a*-suppressed cells as well (Fig. 2C).

### Regulation of cartilage degradation by HIF-1α

**Figure 3:**
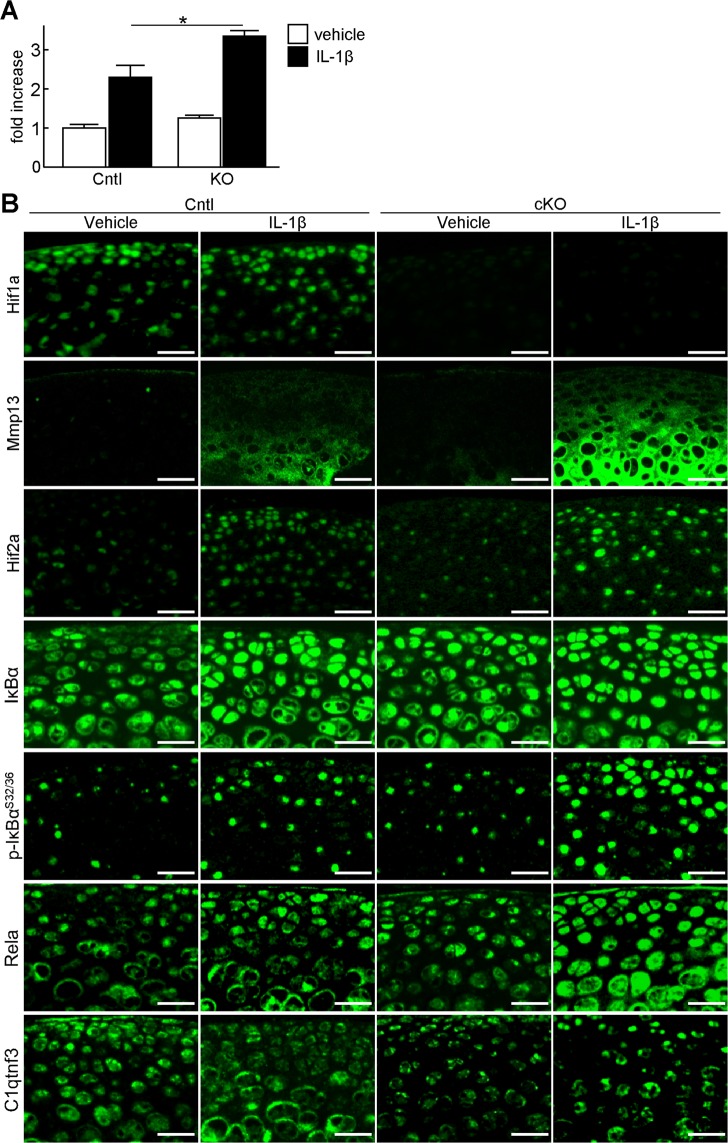
Regulation of cartilage degradation by HIF-1α. (**A**) Amount of glycosaminoglycans released into the medium determined by the dimethylmethylene blue assay during 3 days culture of *Hif1a*^*fl/fl*^ (Cntl) and *Col2a1-Cre*^*ERT2*^*;Hif1a*^*fl/fl*^ (KO) femoral heads treated with or without 10 ng/mL IL-1β under the hypoxic condition (3% O_2_). Tamoxifen induction was performed three days before sacrifice. Bars show the mean ± SD of three samples per group. *P < 0.05. (**B**) Immunofluorescence of Mmp13, Hif2a, and Hif1a in the Cntl and KO femoral head cartilage after 3-day culture with or without 10 ng/mL IL-1β. Scale bars, 50 µm.

To examine whether HIF-1α is involved in cartilage degradation through regulation of HIF-2α and Mmp13, we performed organ culture of mouse femoral heads. We obtained femoral heads from *Col2a1-Cre*^*ERT2*^*; Hif1a*^*fl/fl*^ (KO) and *Hif1a*^*fl/fl*^ (Cntl) mice after tamoxifen treatment, and cultured them with or without IL-1β under hypoxia. The increased amount of glycosaminoglycans released into the medium by IL-1β treatment was further enhanced in the KO femoral head (Fig. 3A). Immunofluorescence using cartilage tissues of these femoral heads showed Mmp13 expression to be markedly enhanced in the KO femoral head treated with IL-1β (Fig. 3B). Since the NF-kB-HIF-2α pathway is a potent regulator of Mmp13 in OA development^8,10^, we analysed expression of the NF-kB-related proteins. In the KO femoral head treated with IL-1β, increase of Rela and phosphorylated IκBα proteins, and nuclear translocation of Rela were more prominent, indicating the enhanced activity of NF-κB signalling pathway (Fig. 3B). The subsequent increase of HIF-2α was also observed (Fig. 3B).

### Regulation of OA development by HIF-1α

**Figure 4:**
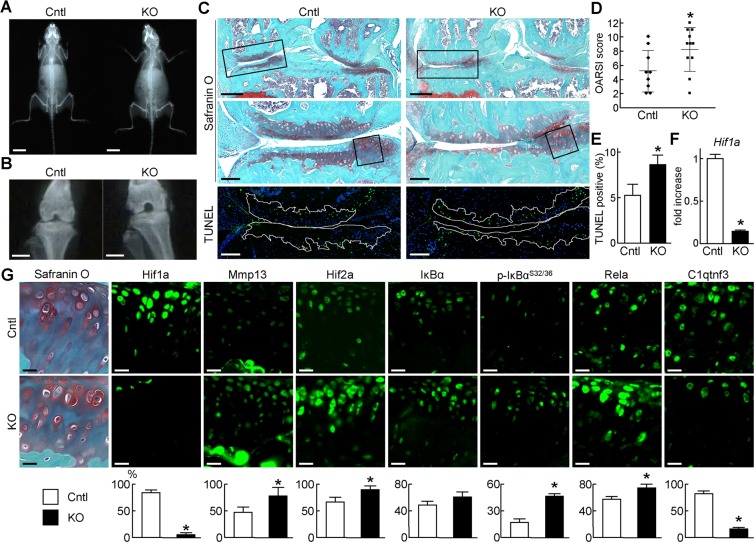
Regulation of OA development by HIF-1α. Plain radiographs of the entire bodies at 8 weeks of age (**A**), and knee joints 8 weeks after OA surgery (**B**) of Cntl and cKO littermates. Scale bars, 10 mm and 1 mm, respectively. (**C**) Safranin O and TUNEL staining’s of Cntl and KO knee joints at 8 weeks after OA surgery (n = 9 and 11, respectively). Tamoxifen induction was performed at 7 weeks. Inset boxes in top images indicate the regions of high magnification Safranin O and TUNEL images. Inset boxes in middle images indicate the regions of immunofluorescence in (**G**). White dotted lines in TUNEL images indicate the cartilage area. Scale bars, 400 µm and 100 µm, respectively. (**D**) OARSI (Osteoarthritis Research Society International) score of OA development. Lines show the mean ± SD. *P < 0.05. (**E**) Rate of TUNEL-positive cells in the cartilage area. Bars show the mean ± SD. *P < 0.05. (**F**) mRNA levels of Hif1a in articular cartilage tissues of Cntl and KO. Bars show the mean ± SD. *P < 0.05. (**G**) Immunofluorescence of Hif1a, Mmp13, Hif2a, IκBα, Ser 32 and 36 dual phosphorylated IκBα, Rela, and C1qtnf3. Scale bars, 20 µm. The percentage of positive cells in the immunofluorescence is shown below. *P < 0.05.

To further elucidate roles of HIF-1α in mouse articular cartilage, we examined OA development of KO and Cntl mice. Tamoxifen induction was performed at 7 weeks. Since the skeletal development was not affected at 8 weeks (Fig. 4A), we created the surgical OA model using KO and Cntl mice. Cartilage degeneration was accelerated in KO knee joints 8 weeks after the surgery, and quantification by the OARSI scoring system confirmed the difference to be statistically significant (Fig. 4B–D). Meanwhile, osteoarthritic changes were not observed in 16-week-old KO and Cntl knee joints without OA surgery (Supplementary Fig. S2). TUNEL staining showed enhanced apoptosis of chondrocyte in KO cartilage (Fig. 4C,E). Efficient knockdown of *Hif1a* in cartilage was confirmed by qPCR and immunofluorescence (Fig. 4F,G). Similar to the results of organ culture, Mmp13 and Hif2a were markedly enhanced by *Hif1a* deletion (Fig. 4G). Immunofluorescence of phosphorylated IκBα and Rela indicated the activation of NF-κB signalling as well (Fig. 4G). Similar expression patterns of marker proteins were observed in 16-week-old mice knee joints without OA surgery (Supplementary Fig. S3). All these data indicate that HIF-1α suppresses the activation of the NF-κB - Hif2a pathway.

### C1qtnf3 mediates downregulation of NF-κB signalling by HIF-1α

**Figure 5:**
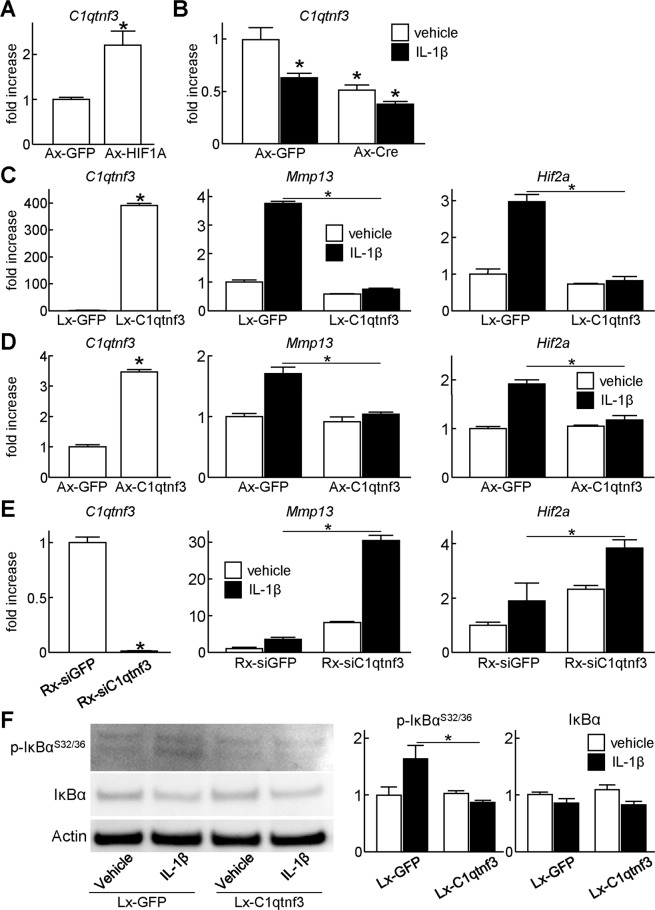
Suppression of catabolic gene expression by C1qtnf3. (**A**) mRNA levels of *C1qtnf3* in WT mouse primary chondrocytes transduced with GFP or HIF1A adenoviral vectors under the hypoxic condition (3% O_2_). GFP or HIF1A was transduced at a multiplicity of infection (MOI) of 100. (**B**) mRNA levels of *C1qtnf3* in *Hif1a*^*fl/fl*^ primary chondrocytes transduced with GFP or Cre adenoviral vectors under the hypoxic condition. GFP or Cre was transduced at a MOI of 100. The cells were treated with or without 10 ng/mL IL-1β for 2 days. (C) mRNA levels of *C1qtnf3*, *Mmp13*, and *Hif2a* in ATDC5 cells transfected via lentivirus with GFP or C1qtnf3. The cells were treated with or without 10 ng/mL IL-1β for 2 days. (**D**) mRNA levels of *C1qtnf3*, *Mmp13*, and *Hif2a* in WT mouse primary chondrocytes transduced with GFP or C1qtnf3 adenoviral vectors at a MOI of 10. The cells were treated with or without 10 ng/mL IL-1β for 2 days. (**E**) mRNA levels of *C1qtnf3*, *Mmp13*, and *Hif2a* in ATDC5 cells transfected via retrovirus with siRNA against GFP or C1qtnf3. The cells were treated with or without 10 ng/mL IL-1β for 2 days. (**F**) Protein levels of IκBα, and Ser 32 and 36 dual phosphorylated IκBα in ATDC5 cells transfected via lentivirus with GFP or C1qtnf3. The cells were treated with or without 10 ng/mL IL-1β for 24 hours. In all panels, bars show the mean ± SD of three samples per group. *P < 0.05.

To investigate how HIF-1α suppresses NF-κB signalling, we performed a microarray analysis using primary *Hif1a*^*fl/fl*^ chondrocytes transduced with adenovirus of GFP or Cre. Lists of upregulated and downregulated genes by *Hif1a* suppression were shown in Supplementary Tables S1 and S2. We further narrowed down NF-κB-related genes by Gene Ontology analysis (Supplementary Tables S3 and S4), and focused on C1q and tumour necrosis factor related protein 3 (C1qtnf3, also known as CTRP3, or CORS-26) among them. C1qtnf3 is a secreted protein structurally closely related to adiponectin, containing an N-terminal collagenous region and a carboxyterminal complement factor C1q globular domain^16^. C1qtnf3 has been known as an anti-inflammatory secreted protein^17^, and a recent study showed that collagen-induced arthritis is exacerbated in *C1qtnf3* null mice^18^. Considering these previous findings, we hypothesized that HIF-1α suppresses NF-κB signalling through C1qtnf3 induction. We first analysed how C1qtnf3 expression was altered by HIF-1α. We found that mRNA level of *C1qtnf3* was significantly increased in the HIF-1α overexpressing cells (Fig. 5A). In contrast, C1qtnf3 was decreased by *Hif1a* knockdown in primary *Hif1a*^*fl/fl*^ chondrocytes, femoral head explants, and articular cartilage of knee joints (Figs. 3B, 4G, 5B, Supplementary Fig. S3). Intriguingly, C1qtnf3 expression was also decreased by the IL-1β treatment (Figs. 3B, 5B).

We then analysed involvement of C1qtnf3 in downregulation of NF-κB signalling and Mmp13 by HIF-1α. We lentivirally generated stable lines of ATDC5 which overexpress GFP or C1qtnf3 dependent on doxycycline induction (Fig. 5C). Expression of *Mmp13* and *Hif2a* were increased by IL-1β treatment in GFP-overexpressing cells, while they were suppressed in C1qtnf3-overexpressing cells (Fig. 5C). Similar results were observed in primary chondrocytes in which C1qtnf3 was overexpressed by adenoviral infection (Fig. 5D). We further generated stable lines of ATDC5 which overexpress siRNA against GFP or C1qtnf3 by retroviral vectors (Fig. 5E). Endogenous *C1qtnf3* expression was efficiently suppressed by stable expression of siRNA (Fig. 5E). Notably, increase of *Mmp13* and *Hif2a* by IL-1β treatment was enhanced by C1qtnf3 suppression (Fig. 5E). We finally examined phosphorylation of IκBα by western blotting (Fig. 5F). Phosphorylated IκBα was upregulated in GFP-overexpressing ATDC5 cells treated with IL-1β; however, it was not increased in C1qtnf3-overexpressing ATDC5 cells by IL-1β treatment (Fig. 5F).

## Discussion

The present study demonstrated that HIF-1α, highly expressed in normal articular cartilage, exerts protective effects against cartilage degeneration and osteoarthritis development. Expression of HIF-2α and activation of NF-κB signalling are negatively regulated by HIF-1α. We further identified C1qtnf3 as a mediator of these anti-catabolic roles of HIF-1α by the microarray analysis. The present data indicate the essential roles of HIF-1α in articular cartilage, which are consistent with a recent study by Bouaziz *et al*.^19^. They have reported that HIF-1α inhibits OA development through suppression of Wnt canonical pathway^19^. HIF-1α knockout leads to increase of Mmp13 and activation of Wnt canonical signalling^19^. They further displayed the direct interaction of HIF-1α and β-catenin, which attenuated transcription factor complex containing β-catenin^19^. Since both up- and down-regulation of Wnt canonical pathway result in acceleration of OA development, its activity should be tightly regulated in articular chondrocytes^3,7^. Considering that HIF-2α enhances the activity of Wnt canonical pathway by interacting β-catenin^20^, balance of HIF-1α and HIF-2α is likely to be the key regulator of Wnt canonical pathway in chondrocytes. Additionally, the present data have demonstrated that HIF-1α suppresses NF-κB signalling, a potent upstream of HIF-2α. Taken together, the anabolic factor HIF-1α and the catabolic factor HIF-2α are complicatedly associated with Wnt and NF-κB signalling pathways.

Under conditions of normoxia, HIF-1α is virtually undetectable due to its rapid degradation through the ubiquitin-proteasome pathway, and has a short half-life (~five minutes)^21,22^. However, when cells are exposed to hypoxia (i.e., 1% O_2_), its half-life is increased to approximately thirty minutes, and exerts cyto-protective effects^21,22^. HIF-1α lability is mediated by the oxygen-dependent degradation domain (ODD)^21^. Although a similar ODD exists in HIF-2α^21^, HIF-2α protein is highly expressed in vascularized tissues^11,23^. The Azo-based fluorescent hypoxic probe indicates that normal articular cartilage is hypoxic, but the oxygen concentration increases along with the OA development (Fig. 1C). The present data is compatible with the previous findings using pimonidazole hydrochloride^19^. Articular cartilage is a large avascular tissue, and is regarded to be nourished from synovial fluid produced by synovial membranes, and partially from subchondral bone by diffusion. In OA joints, synovial membranes usually become hyperplasia, and subchondral bone displays sclerosis. Hypervascularity of synovial membranes may increase the oxygen concentration of synovial fluid, and lead to HIF-1α degradation, which subsequently results in cartilage degeneration through enhanced chondrocyte apoptosis and activation of NF-κB and Wnt canonical signalling pathways.

The roles of hypoxia and HIF proteins in OA have been widely studied using mice models and human surgical samples. A series of previous studies show that HIF-1α induces anabolic genes such as COL2A1 and aggrecan, and plays essential roles in chondrocyte survival^24^. Additionally, Thoms *et al*. reported that hypoxic conditions suppress degradation of human articular cartilage in explant culture^25^. They showed that HIF-1α mediates suppression of the cartilage catabolism under hypoxic conditions, while HIF-2α induces sex determining region Y-box 9 (SOX9), a master transcription factor for chondrogenesis^25^. Although the roles of HIF-2α in human cartilage are still controversial, the hypoxic conditions and HIF-1α protein certainly contribute to cartilage homeostasis.

The previous studies showed severe impairment of joint formation and skeletal growth by HIF-1α deletion in limb bud mesenchyme, and enhanced apoptosis of growth plate chondrocytes in chondrocyte-specific HIF-1α deletion^13,14^. Interestingly, spontaneous OA changes were not obvious in 16-week-old chondrocyte-specific HIF-1α knockout mice which had received tamoxifen injection at 7 weeks (Supplementary Fig. S2). Zhang *et al*. recently showed that when chondrocyte death was induced prior to surgical OA mice models, articular cartilage damage decreased compared to those with intact chondrocytes^26^. They suggested that chondrocyte catabolism, not cell death, contributed to OA development by surgical induction^26^. Considering this theory, HIF-1α deletion in adult articular chondrocytes may not immediately lead to cartilage damage through enhanced apoptosis in the natural course, but may accelerate OA by surgical induction through suppression of anti-catabolism. Meanwhile, the decrease of HIF-1α protein level with aging was rather moderate, compared with that in OA progression by surgical induction (Fig. 1A,B). Further studies are necessary to determine roles of HIF-1α in OA development with aging.

The present study revealed that C1qtnf3, known as anti-inflammatory secreted protein, is induced by HIF-1α, and suppresses NF-κB signalling. C1qtnf3 inhibits LPS-induced inflammatory cytokine production from human adipocytes, monocytes, and fibroblasts^17,27,28^. *C1qtnf3* null mice are fertile, and were born in the expected Mendelian ratios, and grow up normally^18^. In spite of no abnormalities in healthy conditions, arthritis incidence and severity were higher in the *C1qtnf3* null mice when they were treated with type II collagen and complete Freund’s adjuvant^18^. Considering that NF-κB signalling and HIF-2α are deeply involved in rheumatoid arthritis as well as OA^29–32^, C1qtnf3 may regulate the inflammatory arthritis through suppressing the NF-κB - HIF-2α axis, similar to the present findings. On the other hand, molecular mechanisms underlying suppression of NF-κB by C1qtnf3 is still unrevealed. A recent report shows that C1qtnf3 regulates expression of fibroblast growth factor receptor-1 (FGFR1) and FGFR3, and further alters phosphorylation of phosphatidylinositol 3-kinase (PI3K) and Akt^33^. C1qtnf3 may contribute to articular cartilage homeostasis through various kinds of pathways, other than NF-κB. Recently, Fernández-Torres *et al*. reported single nucleotide polymorphisms (SNPs) which are involved both in knee OA development and the HIF-1α signaling pathway^34^. They displayed SNPs of *AKT2*, *IGF1*, *COL2A1*, and *GSK3B*^34^, indicating that HIF-1α may affect OA pathogenesis through various signalling pathways such as insulin growth factor 1 (IGF1) – insulin receptor substrate (IRS) pathway, phosphoinositide 3-kinase (PI3K)–AKT pathway, mammallian target of rapamycin (mTOR), and canonical Wnt pathway, as well as NF-κB.

In conclusion, we demonstrated that HIF-1α is involved in chondrocyte survival in articular cartilage and maintains articular cartilage homeostasis through suppression of the NF-κB - HIF-2α axis. We further identified that C1qtnf3 mediates the cartilage-protective effects of HIF-1α as its downstream molecule. The present data may provide a clue to development of novel therapeutics against OA.

## Methods

### Mice

All experiments were performed according to protocols approved by the Animal Care and Use Committee of The University of Tokyo. In each experiment, we compared the genotypes of littermates maintained in a C57BL/6 J background. *Col2a1-Cre*^*ERT2*^ mice were obtained from Dr. Di Chen^35^. Mice carrying the loxP-flanked conditional alleles of HIF-1α in C57BL/6 background were obtained from Dr. Randall S Johnson^36,37^. *Col2a1-Cre*^*ERT2*^ mice were mated with *Hif1a*^*fl/fl*^ mice to generate *Col2a1-Cre*^*ERT2*^*:Hif1a*^*fl/+*^ and these were mated to *Hif1a*^*fl/fl*^ mice to generate *Col2a1-Cre*^*ERT2*^*:Hif1a*^*fl/fl*^ mice.

### Osteoarthritis experiments

Osteoarthritis was induced by resection of the medial collateral ligament and medial meniscus to *Hif1a*^*fl/fl*^ (n = 10) and *Col2a1-Cre*^*ERT2*^*:Hif1a*^*fl/fl*^ (n = 10) littermates at 8 weeks of age under general anaesthetics^38^. Tamoxifen (100 µg per gram of body weight) was injected intraperiosteally to the mice 1 week prior to surgery for 5 consecutive days. The progression of osteoarthritis in the mice were analysed 8 weeks after surgery.

### Histological analyses

For hypoxic probe experiments, we prepared frozen sections according to Kawamoto’s film method^39,40^. In brief, the mice were sacrificed by cervical dislocation and fixed immediately with 4% paraformaldehyde to terminate the hypoxic reaction. The knee was taken out and placed into hexane with dry ice, and embedded in SCEM compound (Section-lab, Hiroshima, Japan). We prepared 4-μm sections using Cryofilm Type IIC (Section-lab) in the sagittal plane of the medial joint space using a Leica cryostat, and stained with Safranin-O according to the standard protocol. Images of the Safranin-O staining and hypoxic probes were captured and analysed by BZ-X710 microscope system (KEYENCE, Osaka, Japan).

To analyse the progression of osteoarthritis, we used paraffin sections. The mouse knee samples were fixed in 4% paraformaldehyde followed by decalcification in ethylenediaminetetraacetic acid at 37 °C for 5 days and embedded in paraffin. 4 µm coronal sections were stained with Safranin O and evaluated using the Osteoarthritis Research Society International (OARSI) scoring system for the medial compartment. For immunohistochemistry, we incubated the sections with antibodies to HIF1A (1:100; NB100–105, Novus Biologicals), MMP13 (1:100; MAB8887, Merck Millipore), HIF2A (1:100; sc-28706, Santa Cruz Biotechnology)), IκBα (1:100; sc-371, Santa Cruz Biotechnology), p-IκBα^S32/36^ (1:100; sc-101713, Santa Cruz Biotechnology), Rela (1:100; #8242, Cell Signalling Technology), C1qtnf3 (1:100; ab36870, Abcam).

For TUNEL assay, we used an *in situ* Cell Death Detection Kit (Roche) and the number of positive cells in the articular cartilage area were counted using analyser software of BZ-X710 microscope system (Keyence).

### Hypoxic probe

Azo-based fluorescent probe, mono-azo rhodamine (MAR), was developed as previously described^15^. To test the sensitivity of the probe *in vitro*, we cultured primary articular chondrocytes under a cover glass to create an oxygen gradient i.e. oxygen concentration decreases towards the centre. The probe was tested at 1 mM, the concentration used for *in vivo* experiments. We used osteoarthritis model mice to evaluate the hypoxic condition in osteoarthritic cartilage. We performed surgery on 8week-old mice and compared the extent of hypoxia at 0, 4, 8 weeks after surgery by injecting 0.1 ml of 1 mM hypoxic probe 4 hours before sacrifice. To deliver the probe consistently, we incised the skin and exposed the knee before injection and sutured the skin all under general anaesthetics.

### Cell cultures

Primary articular chondrocytes were isolated from 6-day-old neonatal mice as described previously^41^. Isolated chondrocytes were cultured in Dulbecco’s modified Eagle’s medium (DMEM) supplemented with 10% fetal bovine serum (FBS) and 1% penicillin-streptomycin (PS) under the normoxic condition (20% O_2_, 5% CO_2_). For hypoxic culture experiments, the hypoxic condition (3% O_2_, 5% CO_2_) was prepared using nitrogen gas. The mouse chondrogenic cell line ATDC5 (Riken Cell Bank, Tsukuba, Japan) was cultured in DMEM/F12(1:1) with 5% FBS.

### Construction of expression vectors

We amplified the coding sequences of Hif1a and C1qtnf3 from mouse complimentary DNA. These were cloned into pCMV-HA (Clontech). Adenoviral vectors for GFP, Cre, Hif1a and C1qtnf3 were created by the AdenoX Expression System (Clontech). The Tet-On 3G system was used to generate GFP, Hif1a, C1qtnf3 expressing ATDC5 cells introduced via lentiviruses. We transfected si-Hif1a (MSS205124, Invitrogen) and its negative control (#12935200, Invitrogen) using lipofectamine (Invitrogen). piGENEmU6 system (iGENE Therapeutics) was used to construct siRNA vector for the mouse *Hif1a* gene targeting 5′-gcatgaggacgtagaggaagt-3′ (nucleotides 732–752), and the siRNA cassette was transferred into pMx vectors for retrovirus vectors as previously described^42^. Each construct was verified by DNA sequencing.

### RT-qPCR

Total RNA was isolated with an RNeasy Mini Kit (Qiagen). One µg of RNA was of total RNA was reverse transcribed with QuantiTect Reverse Transcription (Qiagen) to prepare single-stranded cDNA. Real time RT- PCR was performed with a Thermal Cycler Dice (Takara) using FastStart Universal SYBR Green Master Mix (Roche) according to the manufacturer’s instructions. The mRNA copy numbers for each gene was calculated using a standard curve generated by serially diluted plasmids contains PCR amplicons synthesized with ZERO Blunt II TOPO Cloning kit (Invitrogen). The copy number was normalized to rodent total RNA (Thermo Fisher Scientific) with mouse RNA polymerase II as the internal control. Primer sequences for real-time RT-PCR are shown in Supplementary Table S5.

### Western blotting

Cells were lysed in M-PER (Thermo-Fisher Scientific) supplemented with COMPLETE protease inhibitor mixture (Roche). Total protein was quantified using the BCA Protein Assay Kit (Pierce). Equal amounts of protein were subjected to SDS-PAGE using 7.5% to 15% Tris-Glycine gradient gels, and blotted onto PVDF membranes (Bio-Rad Laboratories, Inc.). After blocking with 5% skimmed milk, membranes were incubated with primary antibodies against HIF-1α (1:1000, NB100–134, Novus Biologicals), IκBα (1:200; sc-371, Santa Cruz Biotechnology), p- IκBα^S32/36^ (1:200; sc-101713, Santa Cruz Biotechnology) or Actin (1:1000, AC-74, Sigma-Aldrich) in Can Get Signal solution (Toyobo). The membranes were then incubated with HRP-conjugated secondary antibody (Promega), and immunoreactive bands were visualized with ECL plus (GE Healthcare) according to the manufacturer’s instructions. Original images of the immunoblots were shown in Supplementary Fig. S4. For quantification of bands, densitometry analysis was performed using ImageJ software (National Institutes of Health).

### Proteoglycan release assay

Proteoglycan release assay was performed as reported previously^43^. Tamoxifen (100 µg per gram of body weight) was injected intraperiosteally to 2-week-old *Col2a1-Cre*^*ERT2*^*:Hif1a*^*fl/fl*^ and *Hif1a*^*fl/fl*^ mice and the femoral heads were collected after 1 week. These were cultured for three days in DMEM with or without IL-1β under the hypoxic condition (3%). The amount of proteoglycan released in the medium was measured by a colorimetric assay using dimethylmethlene blue.

### Microarray analysis

Total RNA was extracted from primary chondrocytes obtained from *Hif1a*^*fl/fl*^ mice transduced with adenovirus expressing GFP or Cre. Harvested RNA was processed into cDNA and hybridized against SurePrint G3 Human Gene Expression 8 × 60 K v3 microarray chips (Agilent Technologies), following the manufacturer’s instructions. The raw data were deposited in the Gene Expression Omnibus (www.ncbi.nlm.nih.gov/geo/) under accession no. GSE106965.

### Statistical analyses

To assess the statistical significance of experimental data, we used a two-tailed unpaired Student’s *t* test in comparison between two groups, and post hoc testing using Tukey’s method in comparison between multiple groups. *P* values less than 0.05 were considered significant.

## Supplementary information

Supplementary Tables and Figures.
