# Supplementary material for: Hypoxia-inducible factor-1 alpha maintains mouse articular cartilage through suppression of NF-κB signaling
Source: Sci Rep. 2020 Mar 25;10:5425. doi: 10.1038/s41598-020-62463-4 (PMC7096515; doi:10.1038/s41598-020-62463-4)
Supplement: Supplementary file 1 — Supplementary Tables and Figures. [file 41598_2020_62463_MOESM1_ESM.pdf]

Supplementary Tables and Figures

Hypoxia-inducible factor-1 alpha maintains mouse articular cartilage through suppression of NF-κB signaling

Keita Okada[1], Daisuke Mori[1,2], Yuma Makii[1], Hideki Nakamoto[1], Yasutaka Murahashi[1], Fumiko Yano[2], Song Ho Chang[1], Yuki Taniguchi[1], Hiroshi Kobayashi[1], Hiroaki Semba[3], Norihiko Takeda[3], Wen Piao[4], Kenjiro Hanaoka[4], Tetsuo Nagano[5], Sakae Tanaka[1], and Taku Saito[1]*

[1]Sensory & Motor System Medicine, [2]Bone and Cartilage Regenerative Medicine, [3]Department of Cardiovascular Medicine, Graduate School of Medicine, [4]Graduate School of Pharmaceutical Sciences, [5]Drug Discovery Initiative, The University of Tokyo, 7-3-1 Hongo, Bunkyo-ku, Tokyo 113-8655, Japan.

Supplementary Table S1. List of top 20 upregulated genes by Hif1a suppression.

	Gene Symbol	Primary Accession	Ratio (Log2)
1	*Capzb*	ENSMUST00000156760	329.2410894
2	*Mrgprx1*	NM_207540	168.4427933
3	*Hspa1a*	NM_010479	164.1703819
4	*Ccsap*	NM_028536	0.133344883
5	*Hspa1b*	NM_010478	0.098122607
6	*Chmp5*	BC058756	1.224607002
7	*Mapre2*	AK168834	1.309540007
8	*Lce1b*	NM_026822	0.80025078
9	*Hes1*	NM_008235	0.921419485
10	*Klf8*	NM_173780	0.172503255
11	*Gm5409*	NM_001003664	0.018700924
12	*Mical2*	AK163231	0.583762626
13	*Tmem52b*	NM_001081186	0.506554246
14	*Arc*	NM_018790	1.760441917
15	*Pcdh15*	NM_001142746	1.272464622
16	*Trim52*	NM_198601	1.451145865
17	*Raxos1*	AK033044	1.424137795
18	*Lrch1*	AK161894	0.987536741
19	*Gm13269*	AK146025	1.612705923
20	*Cck*	NM_001284508	3.180844733

Supplementary Table S2. List of top 20 downregulated genes by Hif1a suppression.

	Gene Symbol	Primary Accession	Ratio (Log2)
1	*Steap4*	NM_054098	0.008777121
2	*Esm1*	NM_023612	0.003333349
3	*Prkca*	NM_011101	0.002970061
4	*Iigp1*	NM_021792	0.004007835
5	*Col3a1*	ENSMUST00000189818	0.007637118
6	*Adamts9*	NM_175314	0.00818089
7	*Rnf150*	NM_177378	0.009537374
8	*Cxcl5*	NM_009141	0.005705795
9	*Chl1*	NM_007697	0.006527987
10	*Dio2*	NM_010050	0.008743719
11	*Ibsp*	NM_008318	0.006871476
12	*Sipa1l1*	NM_001167983	0.012973994
13	*Rbms3*	NM_001172123	0.012265185
14	*Fpr1*	NM_013521	0.01470344
15	*Cstad*	NM_030137	0.016770423
16	*Mme*	ENSMUST00000193805	0.008609999
17	*Col1a2*	ENSMUST00000148864	0.009917955
18	*Tnfsf11*	NM_011613	0.018984129
19	*Slc35d2*	NM_001001321	0.007078266
20	*Sema5a*	NM_009154	0.010824283

Supplementary Table S3. List of top 10 upregulated NF-κB-related genes by Hif1a suppression.

	Gene Symbol	Primary Accession	Ratio (Log2)
1	*Zc3h12a*	AK163109	5.62595461
2	*Litaf*	NM_019980	2.829985196
3	*Nfkbid*	NM_172142	2.40326852
4	*Prdx2*	AK006748	2.374581213
5	*Siva1*	NM_013929	2.32474428
6	*Irak2*	AK033707	2.25800952
7	*Cyld*	NM_001128171	2.004497765
8	*Pycard*	AK050905	1.86071861
9	*Foxp3*	NM_001199347	1.747680377
10	*Commd6*	NM_001033132	1.592457304

Supplementary Table S4. List of top 10 downregulated NF-κB-related genes by Hif1a suppression.

	Gene Symbol	Primary Accession	Ratio (Log2)
1	*Irg1*	NM_008392	0.029837813
2	*Ppm1b*	NM_011151	0.055997188
3	*C1qtnf3*	NM_030888	0.064561046
4	*Trim21*	NM_009277	0.075364305
5	*Rwdd3*	NM_025637	0.087121002
6	*Cyp1b1*	NM_009994	0.121780415
7	*Arrb1*	NM_177231	0.124198252
8	*Ppm1a*	XM_006515603	0.134266174
9	*Nlrc5*	NM_001033207	0.143212261
10	*Irak3*	NM_028679	0.148257635

Supplementary Table S5. Primers used for RT-qPCR.

gene		sequence (5'-3')
Hif1a	F	tgctcatcagttgccacttc
	R	tgggccatttctgtgtgtaa
Hif2a	F	tgagttggctcatgagttgc
	R	ctcacggatctcctcatggt
Mmp13	F	aggccttcagaaaagccttc
	R	tccttggagtgatccagacc
C1qtnf3	F	tcgatgtcatgactgggaga
	R	tgcatggttgctggatgtat

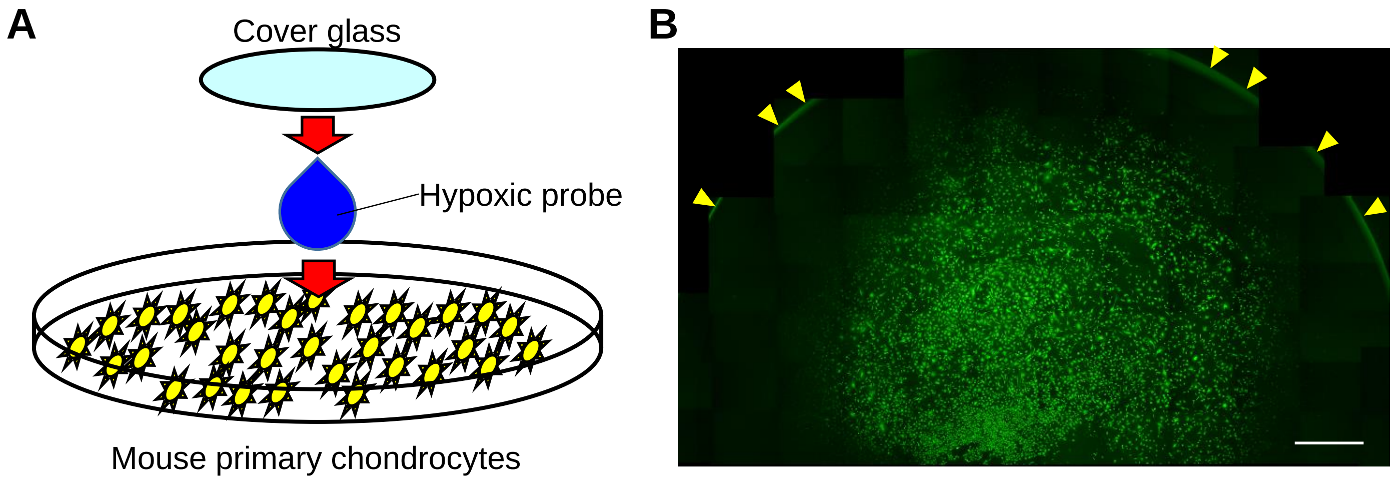

Supplementary Figure S1. Signal intensity of a fluorescent hypoxic probe in mouse primary chondrocytes. (**A**) Schematic of the procedure for testing the hypoxic probe using mouse primary chondrocytes. Cover glass was put onto the cells after the hypoxic probe was added. (**B**) Fluorescent image of the cells one hour after addition of the hypoxic probe. Yellow arrowheads indicate the edge of the cover glass. Scale bar, 1 mm.

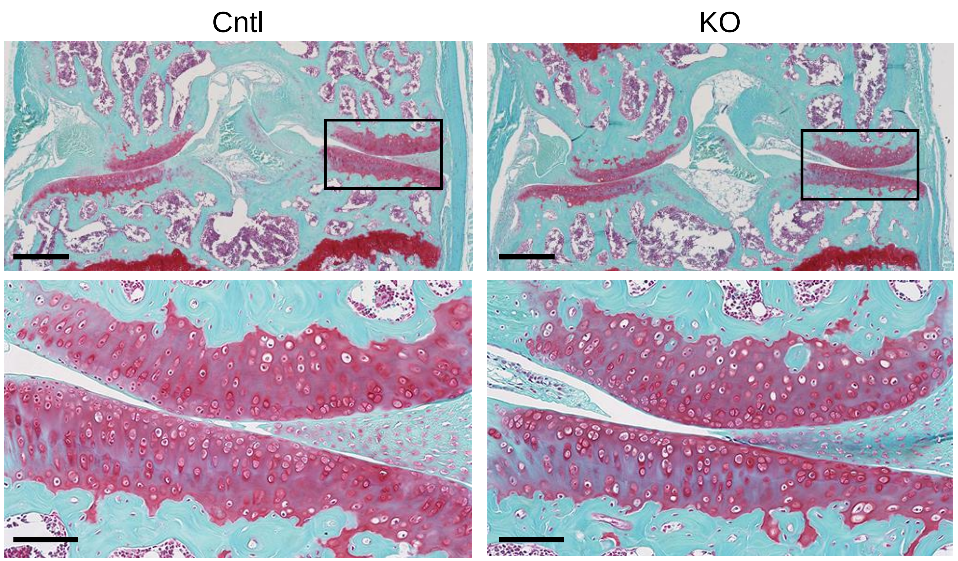

Supplementary Figure S2. Safranin O of articular cartilage of 16-week-old Cntl and KO knee joints without OA surgery. Inset boxes in top images indicate the regions of high magnification Safranin O images. Scale bars, 400 µm and 100 µm, respectively.

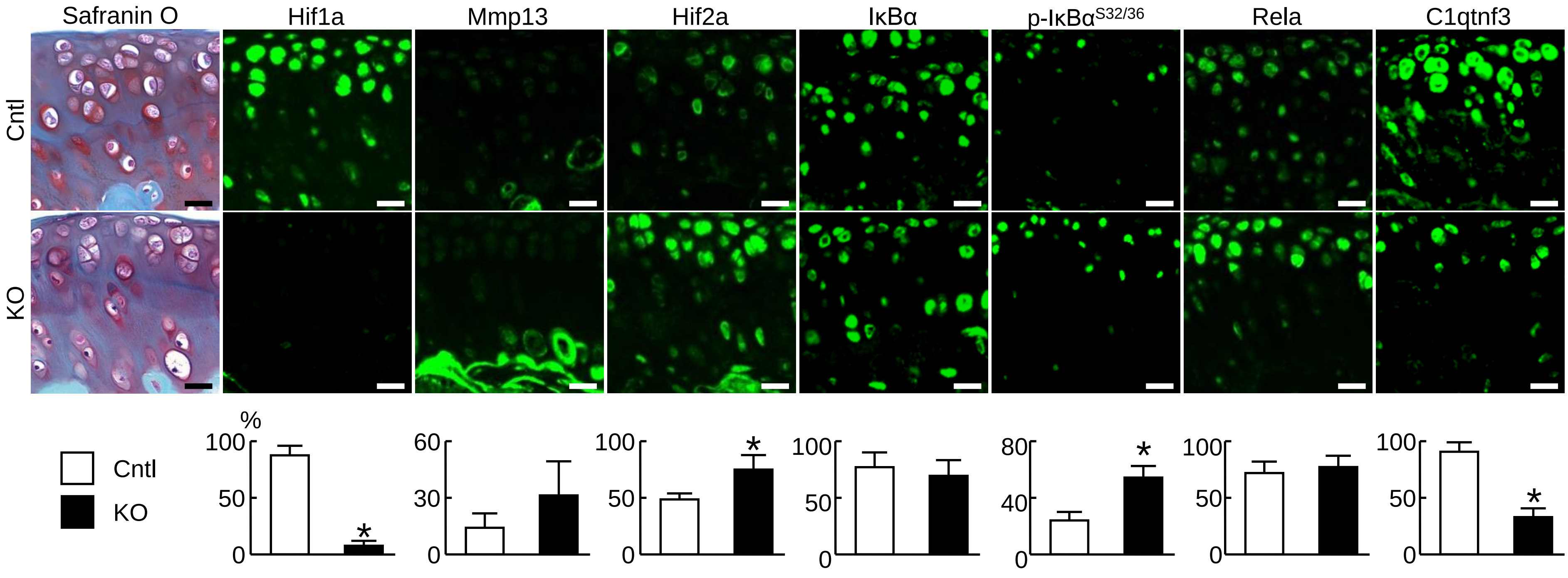

Supplementary Figure S3. Safranin O staining and immunofluorescence of Hif1a, Mmp13, Hif2a, IκBα, Ser 32 and 36 dual phosphorylated IκBα, Rela, and C1qtnf3 in articular cartilage of 16-week-old Cntl and KO knee joints without OA surgery. Scale bars, 20 μm. The percentage of positive cells in the immunofluorescence is shown below. *$P < 0.05$.

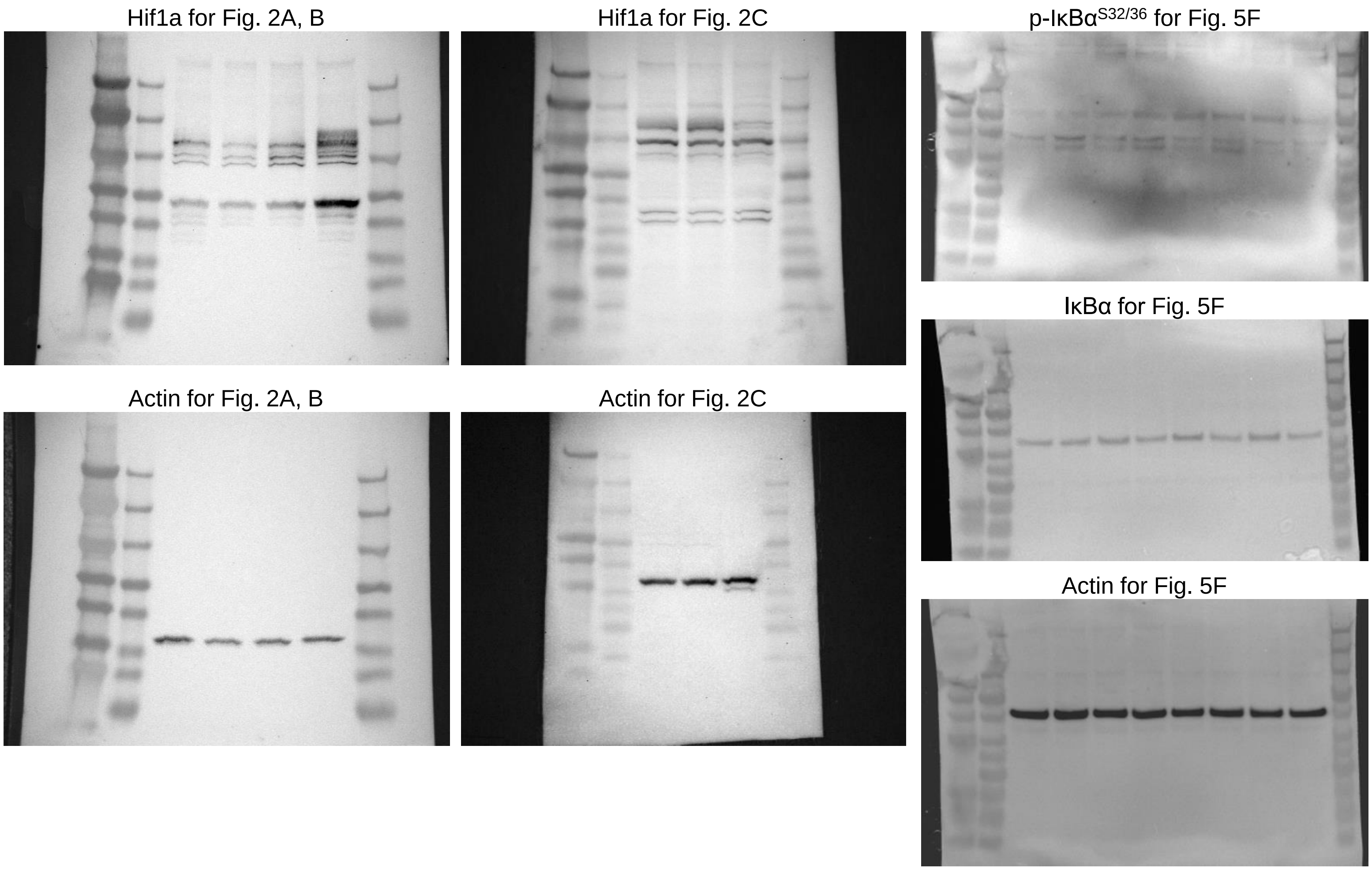

Supplementary Figure S4. Original images of the immunoblots shown in Fig. 2 and 5.
